# A rare pediatric cardiac papillary fibroelastoma: a case report

**DOI:** 10.3389/fcvm.2026.1623898

**Published:** 2026-05-20

**Authors:** Jiayi Lin, Erjia Huang, Wei Li, Yuanyu Zhou, Xiaoqing Wu, Weijian Chen, Wei Su, Xicheng Deng

**Affiliations:** 1 Heart Center, The Affiliated Children’s Hospital of Xiangya School of Medicine, Central South University, Changsha, China; 2 Department of Cardiovascular Surgery, The Second Xiangya Hospital, Central South University, Changsha, China; 3 Department of Pediatrics, Affiliated Hospital of Xiangnan University, Chenzhou, China; 4 Department of Pathology, The Affiliated Children’s Hospital of Xiangya School of Medicine, Central South University, Changsha, China

**Keywords:** echocardiography, papillary fibroelastoma, pediatric cardiac tumor, surgical management, tricuspid valve mass

## Abstract

Cardiac papillary fibroelastoma (PFE) is rare in children, and large (>30 mm), mobile, right-sided PFEs are exceptionally uncommon. This report describes a 7-year-and-6-month-old patient who presented with upper respiratory tract infection and an incidental cardiac murmur. Transthoracic echocardiography revealed a 30 × 25 × 21 mm pedunculated, mobile mass on the anterior tricuspid valve leaflet. Differential diagnosis included thrombus, vegetation, and other cardiac tumors. Surgical excision with tricuspid valvuloplasty was performed due to embolization risk. Gross examination showed a cauliflower-like mass. The patient recovered uneventfully with preserved valve function. This case demonstrates that large, mobile right-sided PFEs can occur in asymptomatic children, and early surgical intervention with valve preservation is recommended to prevent embolic complications.

## Introduction

Cardiac papillary fibroelastomas (PFEs) predominantly occur in middle-aged and elderly individuals. They often first present as thromboembolic events, and typically arising from cardiac valves, particularly the aortic and mitral valves tumors are commonly asymptomatic and identified incidentally during imaging modalities such as echocardiography, chest CT, or cardiac MRI. Morphologically, they present as oval, elliptical, or irregular masses with well-defined borders and a soft texture, attached to the heart via a narrow stalk that dictates their mobility (1, 2). The diagnostic challenge lies in their similarity to thrombi or vegetations, necessitating histopathological confirmation following surgical excision (3).

The early occurrence in childhood and the large tumor size in a pediatric patient highlight the need for systematic evaluation of incidentally detected heart murmurs, even without overt symptoms (4, 5). This case illustrates how proactive diagnostic approaches and tailored treatment protocols can optimize therapeutic outcomes and improve the quality of life for affected children pediatric PFEs have been reported, tumors of this size remain rare and present diagnostic and therapeutic challenges (6, 7).

## Case report

**Figure 1:**
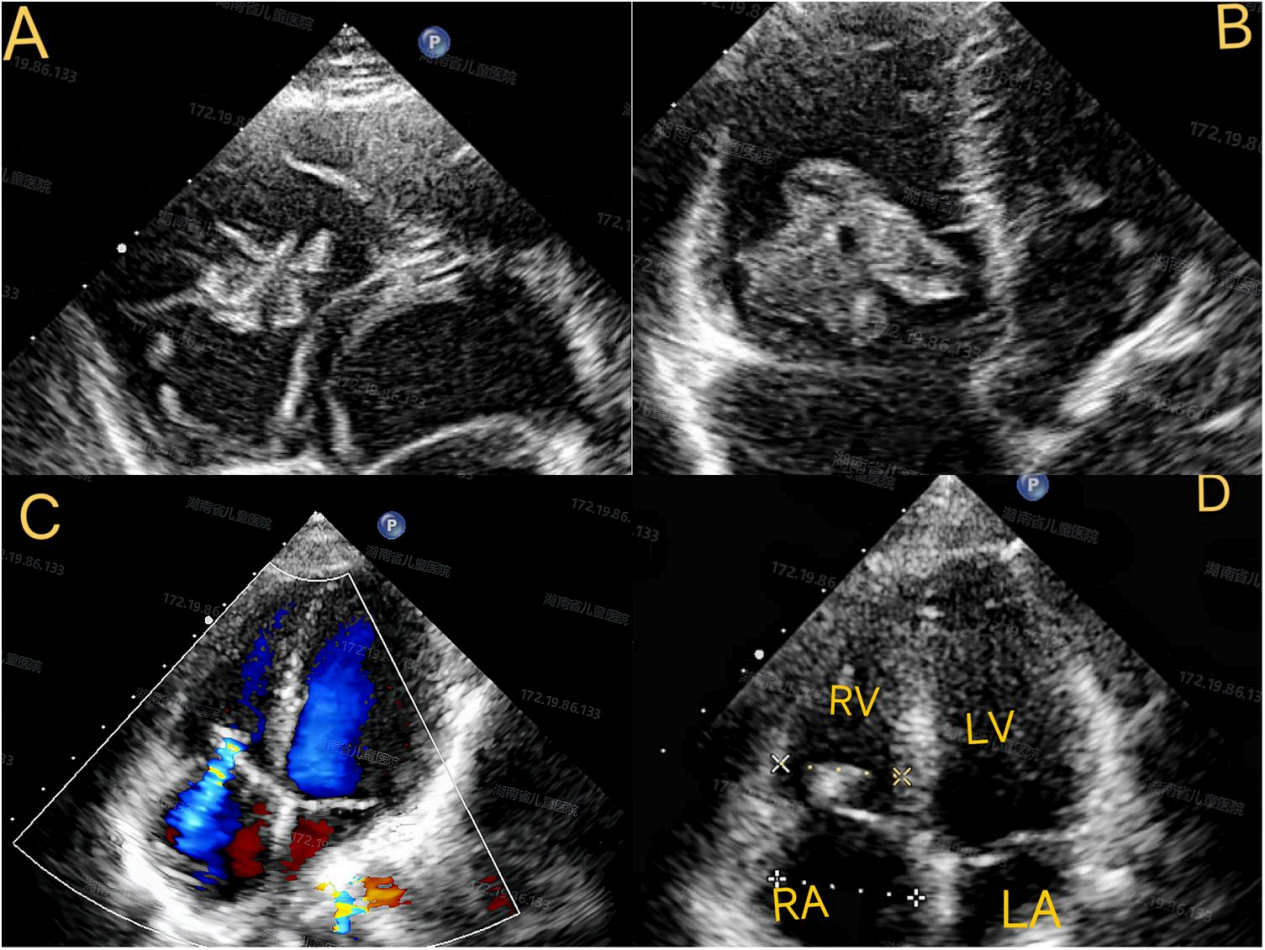
Transthoracic echocardiography images. **(A)** Apical four-chamber view showing a hyperechoic mass (30 × 25 × 21 mm) attached to the anterior leaflet of the tricuspid valve. **(B)** The mass that prolapses into the right ventricle during diastole. **(C)** Transesophageal echocardiography confirming complete excision. **(D)** Postoperative follow-up showing mild tricuspid regurgitation. RA, right atrium; RV, right ventricle; LA, left atrium; LV, left ventricle.

A 7-year-and-6-month-old patient was admitted for symptoms of a cold. Upon physical examination, a cardiac murmur was detected. This murmur was described as a grade 3/6 systolic ejection murmur, with the best auscultation location at the left lower sternal border, without radiation, and without a diastolic component. The patient had no relevant medical history or evidence of infection, trauma, or other triggering factors. Blood cultures were obtained and were negative. Inflammatory markers were mildly elevated, consistent with viral upper respiratory infection but not suggestive of bacterial endocarditis. The criteria for infective endocarditis were not met. However, its distinct characteristics prompted further cardiac evaluation. A transthoracic echocardiogram showed a 30 mm × 25 mm × 21 mm hyperechoic mass with a basal width of 15 mm attached to the anterior to the tricuspid valve (Figures 1A,B). The mass was pedunculated and highly mobile. Real-time imaging confirmed that the mass entered the right ventricle through the tricuspid valve during diastole and returned to the right atrium during systole, with irregular, frond-like edges.

The dimensions of the four cardiac chambers were within normal limits, and the ejection fraction (EF) was 73%. Notably, the patient exhibited no symptoms. However, considering the risk of detachment due to the mass's movement with the cardiac cycle, surgical removal of the mass was decided upon (8). Cardiac magnetic resonance imaging (MRI) was considered but not performed because of the urgency for surgical intervention given the highly mobile large mass and the risk of embolization. Cardiac MRI was considered for tissue characterization but deferred due to: (a) the immediate embolic risk posed by the highly mobile, large mass (>30 mm with >15 mm stalk); (b) surgical indication was established regardless of MRI findings given the size and mobility; and (c) TTE and intraoperative transesophageal echocardiography (TEE) provided adequate anatomical definition for surgical planning (9).

**Figure 2:**
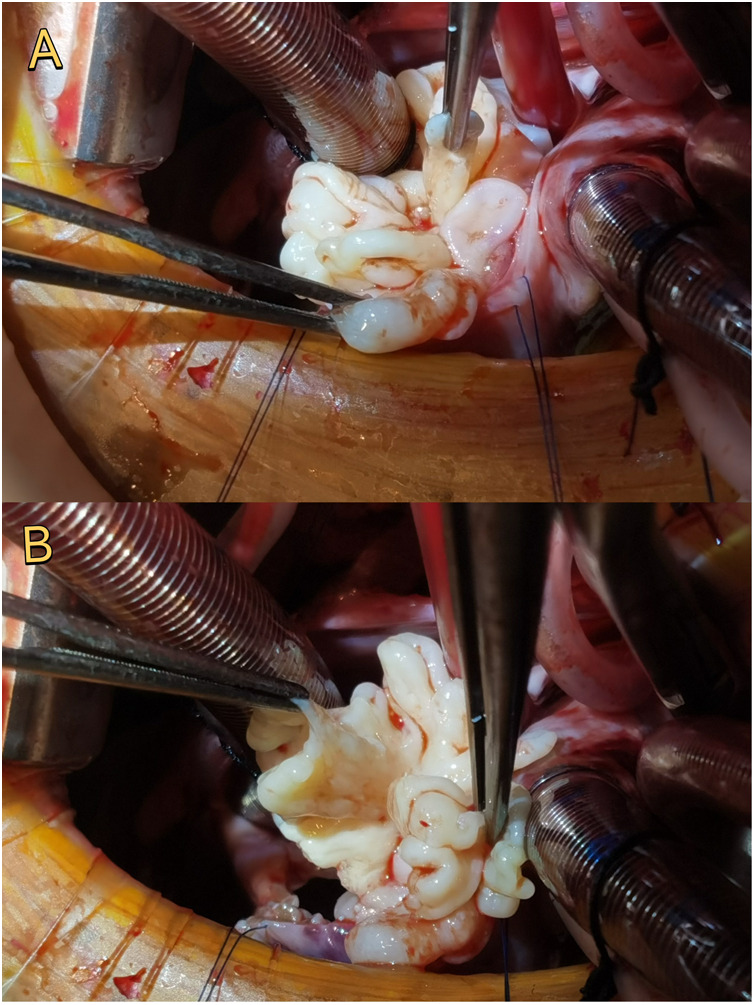
Intraoperative findings. The mass had a cauliflower-like appearance, approximately 30 × 30 × 25 mm, attached to the tip of the anterior leaflet of the tricuspid valve **(A)**. After excision, a small amount of friable tissue was noted at the base; the defect was repaired with interrupted sutures and posterior annuloplasty **(B).**

The surgical approach was through a right infra-axillary incision. Intraoperative exploration revealed a mass approximately 30 mm × 30 mm × 25 mm in size, with a cauliflower-like appearance, attached to the tip of the anterior leaflet of the tricuspid valve and a small amount of friable tissue at the base anterior to the valve tip (Figure 2). The competence of the tricuspid valve was tested by injecting saline into the right ventricle through the valve (float test) after excision, which confirmed significant regurgitation. Therefore, tricuspid valvuloplasty was performed by placing several interrupted figure-of-eight sutures to close the defect created by tumor excision, followed by posterior annuloplasty to reduce the annular size and improve leaflet coaptation. TEE confirmed complete mass removal and normal valve function (Figure 1C).

**Figure 3:**
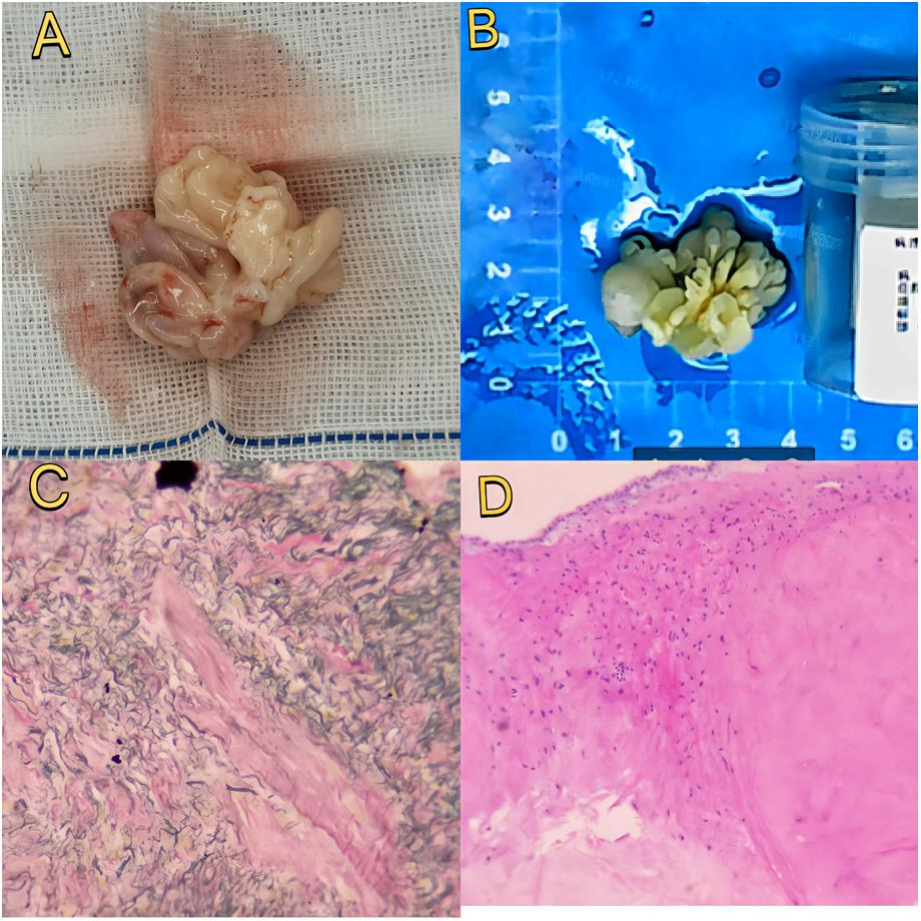
The close-up of the specimen **(A)** and subsequent histopathological analysis confirmed the mass as cardiac papillary fibroelastoma. The submitted specimen is a cauliflower-like tumor, approximately 30 × 20 × 20 mm in size, with a gray-white firm texture, showing cystic changes **(B)**. Subsequent histopathological analysis confirmed the mass as a cardiac papillary fibroelastoma, with the left side being immunohistochemistry **(C)** and the right side being special staining (H&E staining) **(D).**

Histopathological analysis confirmed cardiac papillary fibroelastoma with characteristic avascular papillary fronds lined by endothelial cells (Figure 3). Immunohistochemistry showed: CD34 (positive, indicating endothelial lining), Vimentin (positive, mesenchymal origin), S-100 (positive, likely indicating sustentacular cells or entrapped neural elements rather than neural crest differentiation), DES and SMA (positive in vascular walls), CR (negative), EMA (negative, excluding epithelial tumors). These findings are consistent with previously reported PFE immunophenotypes. The recovery was uneventful, and the patient was discharged one week after surgery. Follow-up examinations (chest x-ray, ECG) showed no abnormalities. Serial echocardiography demonstrated preserved right ventricular function, mild stable tricuspid regurgitation, and no recurrence (Figure 1D). The patient remained asymptomatic with normal exercise tolerance at follow-up.

## Discussion

Cardiac papillary fibroelastoma is a rare benign cardiac tumor, primarily occurring on cardiac valves, especially the left-sided valves. The clinical presentation of cardiac papillary fibroelastoma is nonspecific and may manifest as chest tightness, shortness of breath, and other symptoms. These tumors are typically small and mobile and may potentially cause embolism. Due to the nonspecific nature of its presentation, diagnosis usually requires various imaging and laboratory tests, including echocardiography, CT scans, and sometimes cardiac catheterization (10). Echocardiography is the most commonly used diagnostic tool as it can visualize the location, size, and characteristics of cardiac tumors (11).

To contextualize the rarity of this case, we reviewed previously reported pediatric tricuspid valve PFEs (Table 1). Among the limited published cases with available data, tumor sizes ranged from 5 to 12 mm, with our case representing the largest at 30 mm—exceeding the previously reported maximum by more than twofold. This size, combined with high mobility and right-sided location, posed unique diagnostic and therapeutic challenges.

**Table 1:** Summary of previously reported pediatric tricuspid valve papillary fibroelastoma cases.

Reference	Year	Age	Tumor Size (mm)	Location	Presentation	Treatment	Outcome
Current case	2024	7.5 y	30 × 25 × 21	Anterior leaflet	Incidental murmur (URTI)	Excision + valvuloplasty	Well
Wang et al. (12)	2012	4 y	10 × 8	Anterior leaflet	Incidental murmur	Surgical excision	Well
Karimi et al. (3)	2013	14 y	12 × 10	Septal leaflet	Dyspnea	Surgical excision	Well
Artunduaga et al. (1)	2017	8 mo	8 × 6	Septal leaflet	Incidental finding	Surgical excision	Well
Xu et al. (2)	2013	1 mo	5 × 4	Anterior leaflet	Cyanosis	Surgical excision	Well
Hami et al. (13)	1999	9 y	NR	NR	NR	Surgical excision	Well
Biçer et al. (4)	2025	NR	NR	Multiple valves	NR	Surgical excision	Well

However, differential diagnosis of cardiac masses can be challenging. The primary differential diagnoses include:

1. Thrombus: Typically associated with low cardiac output states, atrial fibrillation, or indwelling catheters. Echocardiographically, thrombi often appear as layered, laminated masses with lower echogenicity and less mobility than PFEs. They may show response to anticoagulation therapy, which PFEs do not.
2. Vegetation (infective endocarditis): Usually associated with fever, positive blood cultures, and valvular destruction. Vegetation tends to be irregular, located on the upstream side of the valve, and may be associated with perivalvular abscesses. In this case, negative blood cultures, absence of sustained fever, and the pedunculated, highly mobile nature of the mass made endocarditis unlikely.
3. Other cardiac tumors: Cardiac fibromas are typically large, solitary, non-mobile masses within the ventricular myocardium, most commonly in the left ventricle, and are often calcified. Rhabdomyomas are multiple, nodular, and commonly associated with tuberous sclerosis. Myxomas are typically located in the left atrium, attached to the interatrial septum.

In contrast, PFEs are small, mobile, valvular tumors with a characteristic frond-like appearance (14, 15). Intraoperative TEE was essential for confirming complete excision and valve competence (16). The treatment of cardiac papillary fibroelastoma generally depends on the patient's symptom severity, tumor size and location, and overall health status. In this case, the tumor was located on the anterior leaflet of the tricuspid valve, and its nature could not be distinguished by preoperative transthoracic echocardiography alone (17). While cardiac MRI with late gadolinium enhancement and tissue characterization sequences can differentiate tumors from thrombi, immediate surgical intervention was warranted in this case due to embolic risk, rendering MRI unnecessary preoperatively. For thrombi, echocardiography frequently discloses a mobile mass accompanied by low cardiac output. Clinical manifestations encompass congestion and a positive response to thrombolytic treatment. For vegetation, echocardiography often shows irregular, mobile vegetation attached to valves, highly associated with infective endocarditis (18).

Regardless, treatment is usually determined by the severity of symptoms, the size and location of the mass, and the patient's overall health status (19, 20). Valve-sparing surgery is paramount in pediatric patients to avoid prosthetic valve replacement and its long-term anticoagulation requirements. In this case, primary repair with annuloplasty successfully restored valve function without residual stenosis or significant regurgitation (21–23).

## Conclusion

Cardiac PFEs, while rare in children, should be considered in the differential diagnosis of incidentally detected cardiac murmurs or right-sided valvular masses. Key clinical messages include: (1) Asymptomatic but mobile PFEs >10 mm in pediatric patients warrant surgical consideration due to lifetime embolic risk; (2) Complete surgical excision with valve preservation is safe and effective, even for large (>30 mm) tricuspid valve tumors; (3) Histopathological examination remains essential for definitive diagnosis, with immunohistochemistry supporting but not replacing morphological assessment.

## Data Availability

The original contributions presented in the study are included in the article/Supplementary Material, further inquiries can be directed to the corresponding authors.
